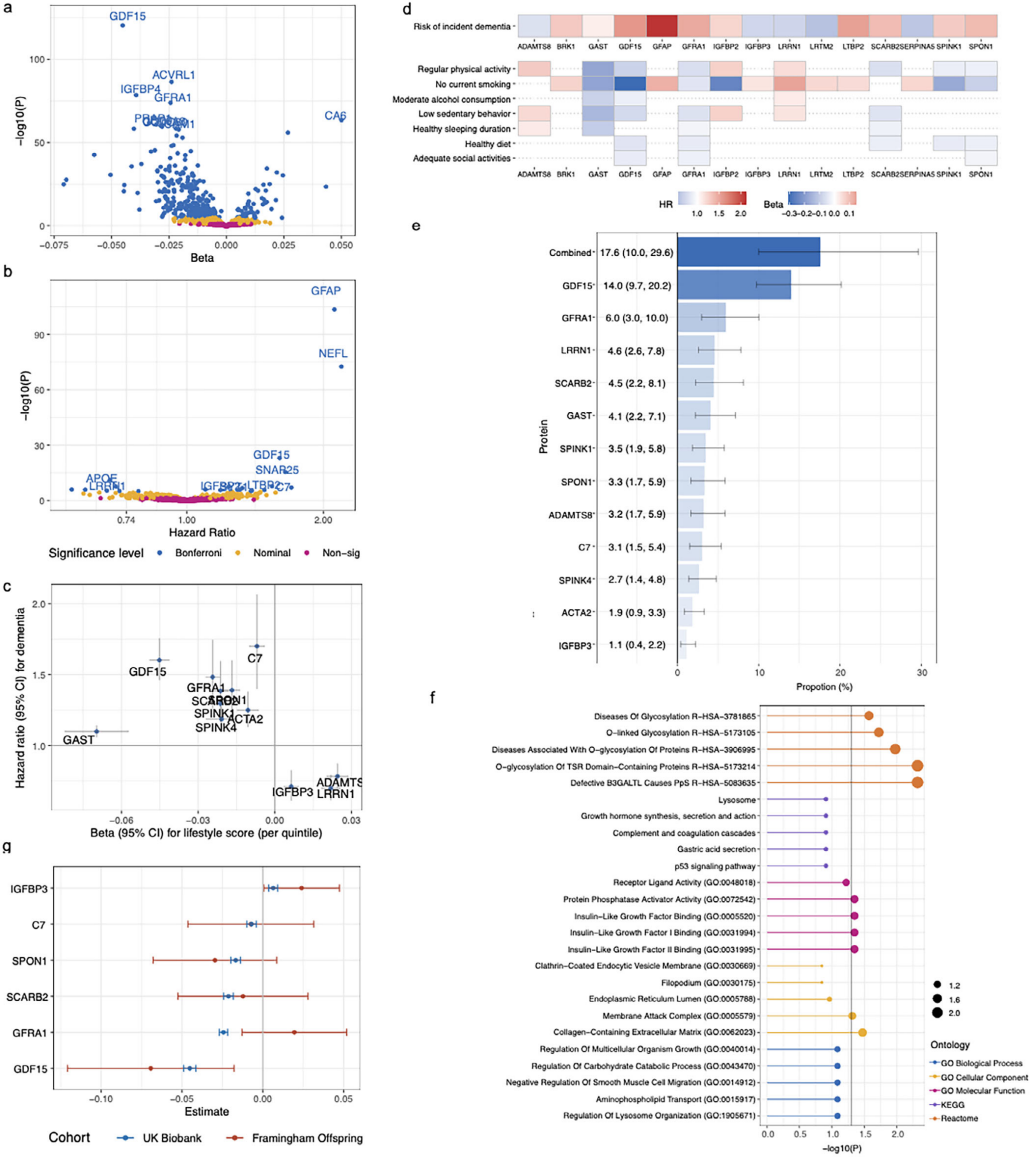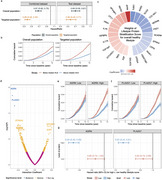# Plasma proteomic profiles mediate and modulate the association between healthy lifestyles and dementia

**DOI:** 10.1002/alz70856_101871

**Published:** 2025-12-25

**Authors:** Jie Shen, Minyu Wu, Yuhui Huang, Minqing Yan, Yang Yang, Xin Xu, Geng Zong, Jie Yang, Xue Li, Hui Chen, Changzheng Yuan

**Affiliations:** ^1^ Zhejiang University School of Medicine, Hangzhou, Zhejiang, China; ^2^ Zhejiang University, Hangzhou, Zhejiang, China; ^3^ School of Public Health, the Second Affiliated Hospital, Zhejiang University School of Medicine, Hangzhou, Zhejiang, China; ^4^ School of Public Health, Zhejiang University School of Medicine, Hangzhou, Zhejiang, China; ^5^ School of Public Health and the Second Affiliated Hospital of School of Medicine, Zhejiang University, Hangzhou, Zhejiang, China; ^6^ CAS Key Laboratory of Nutrition, Metabolism and Food Safety, Shanghai Institute of Nutrition and Health, University of Chinese Academy of Sciences, Shanghai, Zhejiang, China; ^7^ Division of Pharmacoepidemiology and Pharmacoeconomics, Department of Medicine, Brigham and Women's Hospital & Harvard Medical School, Boston, MA, USA

## Abstract

**Background:**

The irreversible nature of dementia necessitates effective lifestyle modifications for primary prevention. High‐throughput proteomics could offer valuable insights into how healthy lifestyles influence cognitive health and provide clues for targeted interventions.

**Method:**

Herein, we conducted comprehensive proteome‐wide association analyses to identify key plasma proteins that mediate the association between an overall healthy lifestyle and the risk of all‐cause dementia. Utilizing data from the UK Biobank, we elucidated the protein mediators of lifestyle‐dementia associations that may serve as intervention intermediate outcomes. Further, we developed and tested a set of lifestyle protein stratification scores (LPSSs) that distinguish targeted populations with high responsiveness of overall and individual lifestyle interventions.

**Result:**

We identified twelve plasma proteomic markers associated with both overall healthy lifestyle score and incident dementia (Bonferroni corrected *P*‐values <0.05) among 32,029 individuals in the UK Biobank. Aggregately, they mediated 17.6% (95% CI, 10.0%, 29.6%) of the lifestyle‐dementia association. The associations between overall healthy lifestyle score and two key proteomic markers (GDF15 and IGFBP3) were externally replicated in a US‐based cohort. Further, the proteome‐wide interaction analysis revealed that the association between lifestyle and dementia risk varied by specific proteomic features. Using a machine‐learning approach, we developed and tested eight LPSSs to identify participants showing stronger associations between overall or individual healthy lifestyles and dementia. Notably, the LPSS for overall healthy lifestyle, comprising 25 proteins, identified a population with a markedly stronger lifestyle‐dementia association (Hazard Ratio: 0.38, 95%CI: 0.32‐0.45) compared with overall population (0.67, 0.60‐0.75). Individuals with higher LPSS may therefore represent a population who may have stronger responses to healthy lifestyle interventions. Targeting interventions to these specific populations may enhance the effectiveness of dementia prevention. For example, the population attributable fraction of an unhealthy diet increased from 1.63% in the overall population to 29.15% in the targeted group with a higher LPSS for a healthy diet.

**Conclusion:**

Our findings indicate that proteomic profiles may serve as intermediate outcomes in lifestyle intervention and facilitate targeted approaches for precision prevention of dementia.